# Comparative anatomy of the passerine carpometacarpus helps illuminate the early fossil record of crown Passeriformes

**DOI:** 10.1111/joa.13761

**Published:** 2022-09-07

**Authors:** Elizabeth M. Steell, Jacqueline M. T. Nguyen, Roger B. J. Benson, Daniel J. Field

**Affiliations:** ^1^ Department of Earth Sciences University of Cambridge Cambridge UK; ^2^ Australian Museum Research Institute Australian Museum Sydney New South Wales Australia; ^3^ College of Science and Engineering Flinders University Adelaide South Australia Australia; ^4^ Department of Earth Sciences University of Oxford Oxford UK; ^5^ Museum of Zoology University of Cambridge Cambridge UK

**Keywords:** carpometacarpus, Passeri, Passeriformes, passerines, songbirds, Tyranni

## Abstract

The hyper‐diverse clade Passeriformes (crown group passerines) comprises over half of extant bird diversity, yet disproportionately few studies have targeted passerine comparative anatomy on a broad phylogenetic scale. This general lack of research attention hinders efforts to interpret the passerine fossil record and obscures patterns of morphological evolution across one of the most diverse clades of extant vertebrates. Numerous potentially important crown passeriform fossils have proven challenging to place phylogenetically, due in part to a paucity of phylogenetically informative characters from across the passerine skeleton. Here, we present a detailed analysis of the morphology of extant passerine carpometacarpi, which are relatively abundant components of the passerine fossil record. We sampled >70% of extant family‐level passerine clades (132 extant species) as well as several fossils from the Oligocene of Europe and scored them for 54 phylogenetically informative carpometacarpus characters optimised on a recently published phylogenomic scaffold. We document a considerable amount of previously undescribed morphological variation among passerine carpometacarpi, and, despite high levels of homoplasy, our results support the presence of representatives of both crown Passeri and crown Tyranni in Europe during the Oligocene.

## Graphical abstract

Here, we bolster knowledge of passerine evolution by presenting a comparative framework for a morphologically variable, functionally important and frequently‐fossilised skeletal component of the wing, the carpometacarpus. We show that some of the earliest crown passerines, including the enigmatic and controversial *Wieslochia weissi*, were crown‐group suboscines—a diverse radiation of extant passerines completely absent from Europe in the present day. Our study constitutes an important first step towards illuminating passerine skeletal evolution by revealing that widespread homoplasy characterises the passerine carpometacarpus.

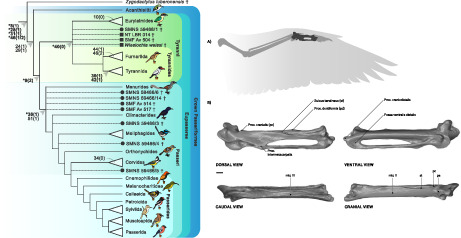

## 1 INTRODUCTION

Passerine birds (Passeriformes) comprise over 6000 extant species, exhibiting a near‐global distribution and exceptional phenotypic, ecological and behavioural disparity. As a result of their extraordinary taxonomic diversity, the evolutionary history of extant passerines has frequently been the subject of large‐scale macroevolutionary studies (e.g., Barker et al., 2004; Day et al., 2020; Derryberry et al., 2011; Moyle et al., 2016; Navalón et al., 2020). However, despite longstanding ornithological interest in passerine evolution, comprehensive characterisations of variation in skeletal morphology across Passeriformes are generally lacking, with most osteological studies on passerines incorporating a scant taxon sample for such a speciose group. This hinders our understanding of major patterns of anatomical evolution across one of the most conspicuous and diverse extant vertebrate clades, as well as our ability to effectively evaluate the passerine fossil record.

Ongoing palaeontological work in Australia and New Zealand has provided important insight into the origins of several crown passerine lineages (Boles, 1995; Nguyen, 2016, 2019; Nguyen et al., 2014, 2016; Worthy et al., 2010), but Paleogene crown passerine fossils from outside Australasia have thus far proven to be more challenging to interpret. As such, with a few notable exceptions (e.g., Bocheński et al., 2018, 2021; Manegold, 2008a, 2008b), the passerine fossil record of Europe has contributed little to our knowledge of the evolutionary origins of most major crown passerine subclades, despite the relative richness of European passerine fossils, some of which represent the oldest known crown passerines (Ballmann, 1969; Manegold, 2008b; Mayr, 2009). In particular, fossil passerines from the Oligocene of Europe (Bocheński et al., 2021; Manegold, 2008b; Mayr & Manegold, 2004, 2006a; Riamon et al., 2020) remain enigmatic. Specimens hypothesised to represent two of the deepest passerine subclades, total‐clade Passeri (“oscines”; Manegold, 2008b) and total‐clade Tyranni (“suboscines”; Bocheński et al., 2021), are known from the European Oligocene (Table 1), yet the precise phylogenetic position of these fossils continues to be controversial. For instance, *Wieslochia weissi* (Mayr & Manegold, 2004) has been alternatively proposed to represent a stem eupasseran (the most exclusive clade composed of Passeri + Tyranni; Mayr & Manegold, 2006b), a stem group representative of Tyranni (Ksepka et al., 2019), and a crown group representative of Eurylaimidae (broadbills, a subclade of Tyranni; Fjeldså et al., 2020). In light of this ongoing phylogenetic uncertainty, reassessing these specimens in additional detail has a potential to significantly clarify a number of outstanding macroevolutionary questions, such as the biogeographic origins of major passerine subclades. For example, Tyranni is entirely absent from the Palearctic in the present day, but firmly establishing the phylogenetic affinities of these Oligocene passerine fossils could support a European origin for the clade.

**TABLE 1:** Oligocene passerine fossils included in the phylogenetic analyses.

Specimen	Age	Preservation	Phylogenetic affinities
*Zygodactylus luberonensis* SMF Av 519	Early Oligocene	Articulated	Stem Passeriformes^a^
Passeriformes sp SMF Av 504	Early Oligocene	Articulated	Total‐clade Tyranni^b^
Passeriformes sp NT‐LBR‐014	Early Oligocene	Articulated	Total‐clade Tyrannida^c^
*Wieslochia weissi* SMNK‐PAL 3980	Early Oligocene	Semi‐articulated	Total‐clade Eupasseres^d^
Passeriformes sp SMNS 59466/1	Late Oligocene	Isolated, 3D	Total‐clade Tyranni^e^
Passeriformes sp SMNS 59466/3	Late Oligocene	Isolated, 3D	Total‐clade Passeri^e^
Passeriformes sp SMNS 59466/4	Late Oligocene	Isolated, 3D	Total‐clade Passeri^e^
Passeriformes sp SMNS 59466/5	Late Oligocene	Isolated, 3D	Total‐clade Passeri^e^
Passeriformes sp SMNS 59466/6	Late Oligocene	Isolated, 3D	Total‐clade Passeri^e^
Passeriformes sp SMNS 59466/14	Late Oligocene	Isolated, 3D	Total‐clade Passeriformes^e^
Passeriformes sp SMF Av 514	Late Oligocene	Isolated, 3D	Total‐clade Passeri^e^
Passeriformes sp SMF Av 517	Late Oligocene	Isolated, 3D	Total‐clade Passeriformes^e^

*Note*: Phylogenetic affinities reflect the conclusions drawn in the original fossil descriptions.

^a^ Mayr (2008);

^b^ Mayr and Manegold (2006a);

^c^ Riamon et al. (2020);

^d^ Mayr and Manegold (2006b);

^e^ Manegold (2008b)

Several morphological synapomorphies have been proposed to diagnose the three deepest extant passerine subclades—Acanthisitti, Passeri and Tyranni (Manegold, 2008b; Mayr & Manegold, 2006b; Worthy et al., 2010). However, most major subclades within these groups remain uncharacterised by discrete synapomorphies or diagnostic character combinations, and few detailed anatomical character‐taxon matrices exist for crown Passeriformes. A frequent misconception that may partly account for this dearth of research attention is the notion that passerine skeletons are generally ‘uniform’ (Mayr, 2009, 2013). However, a greater impediment to research may simply be the sheer taxonomic diversity of Passeriformes combined with uncertainty regarding the higher‐order phylogenetic relationships of passerines that have only recently been resolved (Harvey et al., 2020; Moyle et al., 2016; Oliveros et al., 2019). The enormous diversity of extant passerines presents a legitimate logistical challenge that has dissuaded attempts to generate large‐scale morphological datasets for crown Passeriformes.

**FIGURE 1:**
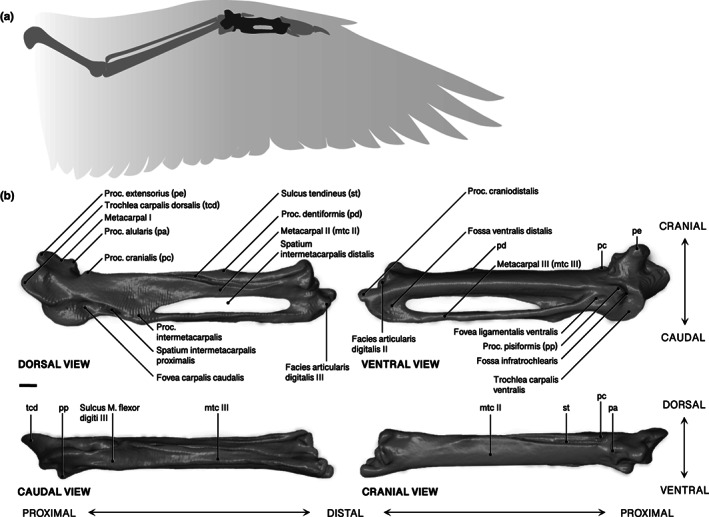
(a) Diagram of an articulated passerine left wing in ventral view with carpometacarpus indicated in dark grey. (b) Labelled right carpometacarpus of *Tyrannus tyrannus* FMNH 487521 in dorsal, ventral, caudal and cranial views. Scale 1 mm.

Here, we broach this gap in our understanding of passerine comparative morphology by focusing on the carpometacarpus. As a comparatively robust component of the wing skeleton (Figure 1), carpometacarpi exhibit reasonable preservation potential in the fossil record (Ballmann, 1969; Manegold, 2008b; Nguyen et al., 2016), and the impressive morphological variability of the carpometacarpus may have phylogenetic relevance. Although carpometacarpi have provided phylogenetically‐informative morphological characters in previous investigations of fossil passerines, available character data for passerine carpometacarpi tend to be either diagnostic only for the deepest clades within crown Passeriformes (Mayr & Manegold, 2006a, 2006b; Manegold, 2008b; Worthy et al., 2010), or for a small number of family‐level passerine subclades (Nguyen et al., 2016; Tomek & Bocheński, 2000). In most cases, the phylogenetic information extracted from isolated fossil passerine carpometacarpi has been limited to the ordinal or subordinal level by a lack of adequate comparative data from across passerine diversity. An in‐depth analysis of morphological variation of the passerine carpometacarpus therefore has potential to inform patterns of morphological evolution and trait acquisition across passerine phylogeny.

We have assembled a large‐scale comparative morphological dataset of passerine carpometacarpi using X‐ray micro‐computed tomography (μCT) in order to evaluate patterns of morphological evolution across Passeriformes. Our taxon sample covers >70% of passerine family‐level diversity, incorporating 132 extant species representing 101 family‐level passerine clades and 10 non‐passerine outgroups. By combining novel osteological characters and re‐evaluated formulations of previously published characters, we have generated a detailed character matrix for the passerine carpometacarpus that incorporates numerous taxa that have never previously been the subject of osteological study. Our character list is fully illustrated with high‐resolution images of carpometacarpi, with the aim of providing a reference for future comparative studies, including evaluations of fossil passerines. We also include several important fossil specimens from the Oligocene of Europe (Table 1) into our phylogenetic analyses, including some of the earliest known crown passerines, most of which have never previously been subjected to quantitative analyses. Our work follows recent advances in resolving the higher‐order phylogenetic interrelationships of passerines from large‐scale phylogenomic analyses (Harvey et al., 2020; Moyle et al., 2016; Oliveros et al., 2019), and we use this emerging phylogenetic framework to map character state transitions across the passerine crown group and identify diagnostic character combinations for major passerine subclades. This study seeks to illuminate the early fossil record of crown passerines by analysing the phylogenetic affinities of European Oligocene fossils, and our carpometacarpus dataset constitutes a first step towards clarifying the evolutionary origins of several major passerine subclades.

## 2 METHODS

Specimens from extant taxa used in this study are registered in the following museum collections: University of Cambridge Museum of Zoology (UMZC), Natural History Museum, UK (NHMUK), Oxford University Museum of Natural History (OUMNH), Field Museum of Natural History (FMNH), Yale Peabody Museum (YPM), Australian Museum (AM), Museums Victoria (NMV) and Australian National Wildlife Collections (ANWC). Digital 3D models were generated through μCT scanning of UMZC, NHMUK, OUMNH, FMNH and YPM skeletal, whole spirit and study skin specimens. Details of specimens used are in Table S1. μCT scans were segmented in VGSTUDIO MAX V3.3. and AvizoLite V9.3. 3D meshes were visualised and studied in Meshlab (Cignoni et al., 2008). CT image volumes and 3D surface models of carpometacarpi are available inMorphosource, and links to these media are provided in Table S1.

Phylogenetic relationships and classification follow Oliveros et al. (2019). Additional clade names taken from Fjeldså et al. (2020) are: Acanthisitti (Acanthisittidae), Menurides (Menuridae + Atrichornithidae), Climacterides (Climacteridae + Ptilonorhynchidae), Meliphagides (Maluridae + (Dasyornithidae + (Meliphagidae + (Pardalotidae + Acanthizidae)))), Orthonychides (Orthonychidae + Pomatostomidae), Callaeida (Callaeidae + Notiomystidae) and Petroicida (Petroicidae + Eupetidae).

Anatomical terminology follows Baumel and Witmer (1993). The following terms are also included: ‘processus dentiformis’ (Lambrecht, 1914), ‘fovea ligamentalis ventralis’ (Livezey & Zusi, 2007), and ‘processus cranialis’ (Manegold, 2008b). We also add two modified terms: ‘fossa distalis ventralis’ and ‘processus craniodistalis’, to clarify the ‘distal fossa’ and ‘finger‐like process’ identified by Mourer‐Chauviré et al. (1989). See Figure 1 for all anatomical features discussed in this study.

**FIGURE 2:**
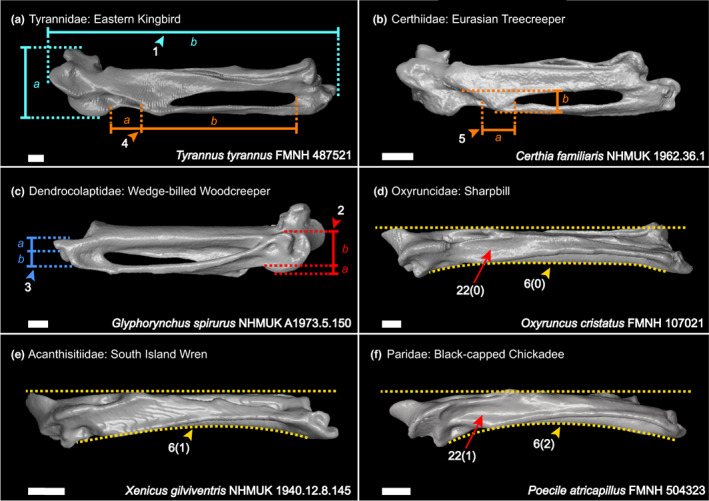
Right carpometacarpus in dorsal (a, b), ventral (c) and caudal (d–f) views. Characters of the whole carpometacarpus (char. 1, *a*:*b*; cyan), trochlea carpalis ventralis and sulcus for M. flexor digiti III (chars. 2, *a*:[*a* + *b*], & 22; red), distal metacarpal III (char. 3, *a*:[*a* + *b*]; blue), proc. intermetacarpalis (chars. 4, *a*:[*a* + *b*] & 5, *a*:*b*; orange) and dorsoventral curvature of the carpometacarpus (char. 6; yellow). Scale 1 mm.

**FIGURE 3:**
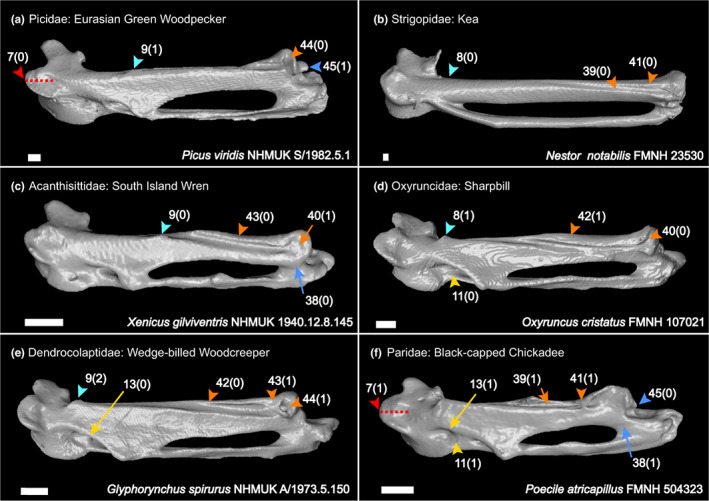
Dorsal view of right carpometacarpus, characters of trochlea carpalis dorsalis (char. 7; red), proc. cranialis (chars. 8 & 9; cyan), fovea carpalis caudalis (chars. 11 & 13; yellow), sulcus tendineus (chars. 39–44; orange) and distal carpometacarpus (chars. 38 & 45; blue). Scale 1 mm.

**FIGURE 4:**
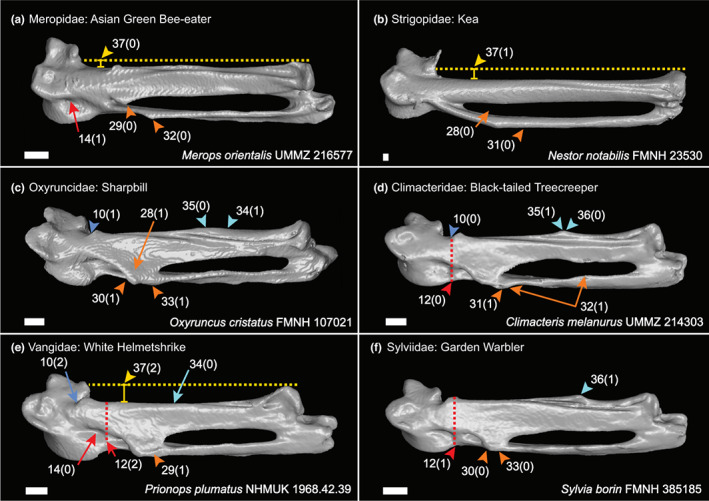
Dorsal view of right carpometacarpus, characters of the proc. cranialis (char. 10; blue), fovea carpalis caudalis (chars. 12 & 14; red), proc. intermetacarpalis (chars. 28–33; orange), os metacarpale II (chars. 34–36; cyan) and distal carpometacarpus (char. 37; yellow). Scale 1 mm.

**FIGURE 5:**
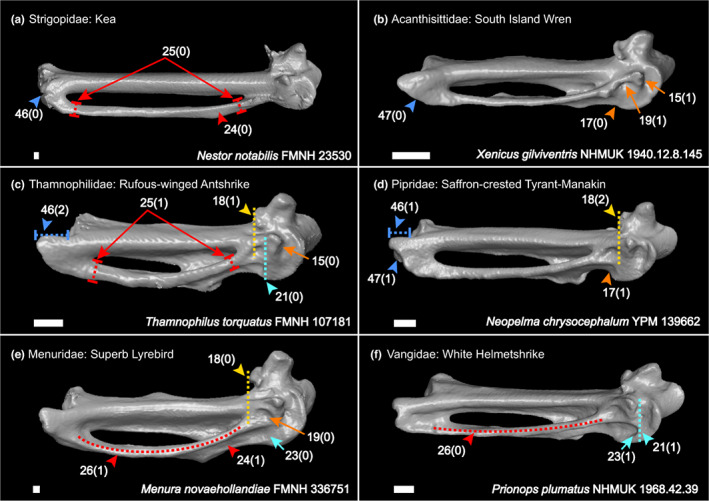
Ventral view of right carpometacarpus, characters of the trochlea carpalis ventralis (chars. 15, 17 & 19; orange), proc. alularis (char. 18; yellow), sulcus for M. flexor digiti III (chars. 21 & 23; cyan), os metacarpale III (chars. 24–26; red) and distal carpometacarpus (chars. 46 & 47; blue). Scale 1 mm.

**FIGURE 6:**
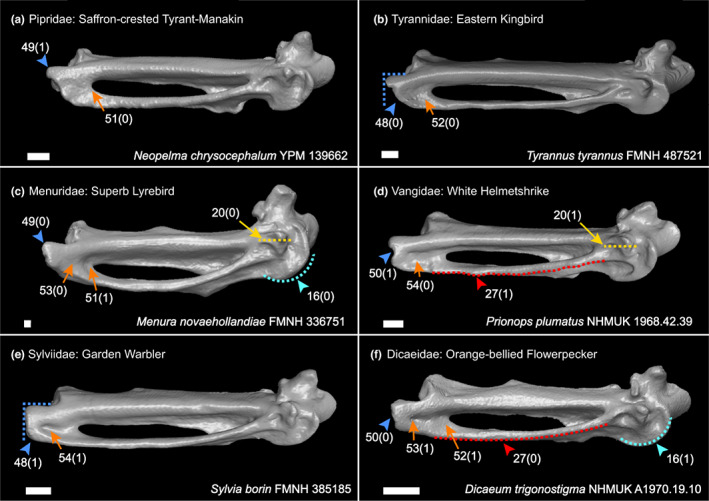
Ventral view of right carpometacarpus, characters of the trochlea carpalis ventralis (char. 16; cyan), fovea lig. entralis (char. 20; yellow), os metacarpale III (chars. 27; red), distal carpometacarpus (chars. 48–50; blue) and fossa distalis ventralis (chars. 51–54; orange). Scale 1 mm.

We generated a character matrix of 54 morphological characters of the passerine carpometacarpus (Figures 2, 3, 4, 5, 6), comprising both previously published and novel characters. Previously published characters were re‐evaluated, and new characters were described from comparisons of 3D digital specimens and skeletal specimens. See Table S2 for the full character list. Taxon sampling generally followed the major passerine subclades sampled in the phylogenomic study of Oliveros et al. (2019). We included 132 extant passerines from 101 families recognised by the phylogenetic study of Oliveros et al. (2019), covering >70% of passerine family‐level diversity. We also included 11 fossil carpometacarpi, see Table 1. These Oligocene fossils were selected based on their morphology being clearly and adequately documented in published literature with good quality photos available for character scoring, and our interest in characterising anatomical transitions relevant to the deepest splits within Passeriformes. We included as many of the earliest crown passerine fossils as possible (early Oligocene in age) that fit the criteria outlined above, as well as several isolated late Oligocene carpometacarpi that are morphologically distinct. We regret that we could not include several specimens of significant interest (described by Bocheński et al., 2011, 2013, 2018, 2021) based on their carpometacarpi being poorly preserved or not clearly discernible in published images.

Character optimisation was carried out under parsimony using the Willi Hennig Society version of TNT (Goloboff et al., 2008) with character state transitions mapped to the Oliveros et al. (2019) topology for Passeriformes, with the addition of the Harvey et al. (2020) topology for intrafamilial relationships within Tyranni and the Prum et al. (2015) topology for relationships between outgroups (molecular scaffold). Additionally, *Zygodactylus luberonensis* SMF Av519 (Mayr, 2008) was included in our molecular scaffold as the sister taxon to crown Passeriformes, based on its phylogenetic position and supporting morphological synapomorphies described by Mayr (2008). Several characters with more than two states were treated as ordered in cases where it was logical to assume that anatomical transitions were likely to pass through separately identified intermediate states (see Table S2). Scripts for all analyses are in Supporting Information.

We conducted four Bayesian phylogenetic analyses to evaluate the affinities of fossil carpometacarpi that were carried out in MrBayes V3.2.2 (Ronquist et al., 2012) using the CIPRES Science Gateway (Miller et al., 2010). We excluded characters 1–5 which are continuous and therefore could not be included. All MrBayes analyses were performed for two runs with four chains and 30 million replicates sampled every 4000 generations with a chain temperature of 0.1 and burn‐in set to 0.25. The likelihood model priors were sampled from a gamma distribution with four rate categories with variable coding for morphological data. Analyses 1–4 were summarised as 50% majority rule trees (contype = Halfcompat). The molecular scaffold (see above) was applied to all extant taxa, and *Zygodactylus luberonensis* was also topologically constrained as the sister group to crown Passeriformes. MrBayes constraint scripts were generated using the package *Paleotree* (Bapst, 2012) in *R* V4.1.1 (R Core Team, 2021).

**FIGURE 7:**
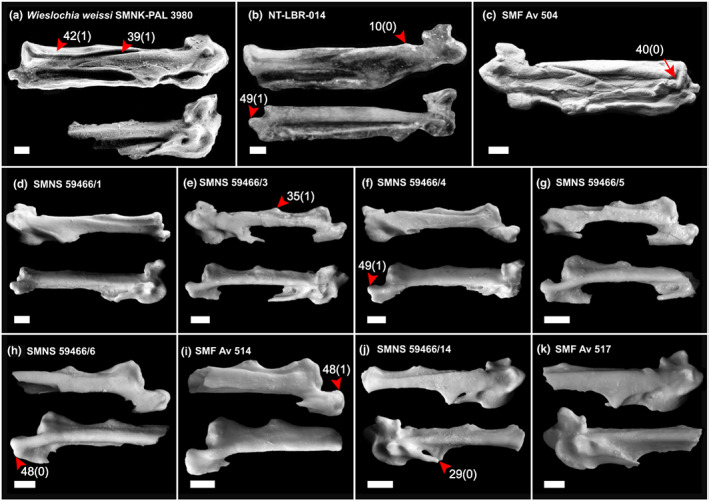
Fossil passerine carpometacarpi examined in this study and included in our phylogenetic analyses. (a) Dorsal view of left carpometacarpus and ventral view of right carpometacarpus of *Wieslochia weissi* (SMNK‐PAL 3980), modified from Mayr and Manegold (2006b). (b) Dorsal view of left carpometacarpus and ventral view of right carpometacarpus of NT‐LBR‐014, modified from Riamon et al. (2020). (c) Dorsal view of right carpometacarpus of SMF Av 504, modified from Mayr and Manegold (2006a). (D–I) dorsal and ventral views of right carpometacarpi of SMNS 59466/1, 59466/3, 59466/4, 59466/5, 59466/6 and SMF Av 514, modified from Manegold (2008b). (J, K) dorsal and ventral views of left carpometacarpi of SMNS 59466/14 and SMF Av 517, modified from Manegold, 2008b. Red arrows indicate important character states discussed in the main text. Scale 1 mm. All images used with permission.

Analysis 1 included all fossils in Table 1 (Figure 7) unconstrained, except *Zygodactylus* (see above), in addition to the full sample of topologically constrained extant taxa. Analyses 2–4 were the same as analysis 1; however, they each included only one of the following fossils: SMNS 59466/3–5 (Table 1), with the following fossils excluded from the analyses: SMNS 59466/6 & 14, SMF Av 514 & 517 (Table 1). The latter fossils were particularly unstable, possibly due to missing data as a consequence of their fragmentary nature causing node collapse in different areas within Passeri; as such, these were excluded from further analyses.

Two additional analyses were performed to evaluate whether inferences from our morphological carpometacarpus dataset could replicate the phylogenetic relationships recovered from the aforementioned molecular datasets; see Supporting Information for methods and results (Figures S5 and S6) of additional analyses.

## 3 RESULTS

### 3.1 Diagnostic character combinations of major passerine subclades

**FIGURE 8:**
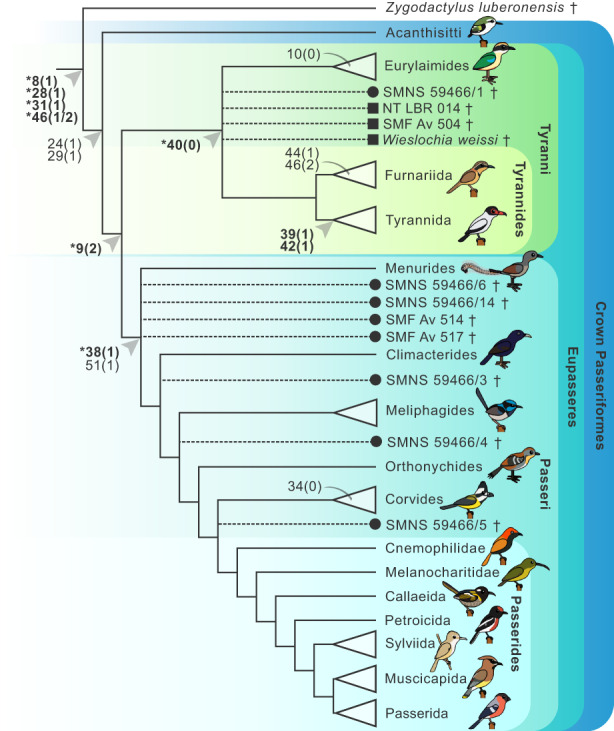
Cladogram summarising results of a Bayesian phylogenetic analysis of the matrix generated in this study, including estimated placement of Oligocene passerine fossils (dotted lines). Fossils are illustrated within an extant clade when support for that position exceeded 0.90 BPP. Early Oligocene specimens are denoted by a circle and late Oligocene specimens denoted by a square. Arrows indicate key character states for nodes. Synapomorphies are highlighted in bold with unreversed synapomorphies denoted by an asterisk. Character states associated with arrows that are not in bold are near‐ubiquitously present for the clade. A curved line indicates notable character states occurring among a subset of taxa within the subclade. Illustrations courtesy of Albert Chen, used with permission.

Diagnostic character combinations for selected passerine subclades are described below. We present unreversed synapomorphies (denoted by ‘*’) for clades recovered from our character state optimisation analysis. That is, character states that are: (i) ubiquitously present (present in all sampled taxa in a clade), (ii) optimise as synapomorphies for the ancestral phylogenetic node of that clade and (iii) are absent in the ancestral phylogenetic node of the immediate sister clade. Additionally, we identify character states that do not fit all criteria outlined above, but which may be useful inclusions for diagnostic character combinations. Characters present through retention (i.e. retained plesiomorphies; that are present for rootward nodes) are denoted by ‘§’. We did not recover any truly unique unreversed synapomorphies for major passerine subclades, although unique synapomorphies within subclades are mentioned. For hierarchically nested clades, characters mentioned for rootward nodes are included in the combination for the node in question. Notable character states are highlighted in Figure 8. Refer to Supporting Information for character optimisation trees and diagnostic combinations for additional passerine subclades (Acanthisitti; Meliphagides; Sylviida; Muscicapida + Passerida; Muscicapida; Passerida).

*Total‐clade Passeriformes*: Cranial process present (*char. 8[1]; Figure 3). Intermetacarpal process present (*char. 28[1]; Figure 4). One or multiple protrusions present on dorsal edge of metacarpal III (*char. 31[1]; Figure 4). Cranial margin of sulcus tendineus delimited or partially delimited by a clearly discernible ridge (char. 41[1]; Figure 3); although note this is absent in *Melanopareia torquata* and char. 41(1) did not optimise as a synapomorphy due to both states occurring in sequential sister taxa, resulting in ambiguity at the Psittacopasseres node. Metacarpal III extending beyond metacarpal II distally (*char. 46[1/2]; Figure 5). For characters 8, 28, 31 and 46, the same states occurred independently in the piciforms and coraciiforms sampled (*Picus viridis*, *Capito niger* and *Merops orientalis*), and character 41(1) is independently present in piciforms, falconiforms (*Falco sparverius* and *Micrastur ruficollis*) and the trogon *Trogon melanurus*.

*Crown Passeriformes*: Ventral orientation of proximal part of metacarpal III so caudal surface is partially visible in ventral view (char. 24[1]; Figure 5), also present in *Columba livia*, but notably absent in the piciform and coraciiform taxa sampled. *Hirundo rustica* was the only crown passeriform investigated in which this feature was absent, which may be linked to specialised flight adaptations in swallows (Passeri: Hirundinidae). Fusion of intermetacarpal process to metacarpal III (char. 29[1]; Figure 4), also observed in piciforms. None of these characters optimised as synapomorphies due to missing data from *Zygodactylus* resulting in ambiguity at the total‐clade Passeriformes node.

*Eupasseres*: Cranial process proximal relative to approximate midpoint of intermetacarpal process (*char. 9[2]; Figure 3), independently present in the barbet *Capito niger*.

*Tyranni*: Trochlea carpalis ventralis is oblong in shape (§char. 16[0]; Figure 6). Dorsal surface of caudodistal region of metacarpal II protrudes dorsally (§char. 38[0]; Figure 3). Distal end of sulcus tendineus does not broaden into a fossa (*char. 40[0]; Figure 3), occurs independently multiple times in some non‐passerines and across Passeri. Distinct process on facies articularis digiti II absent (§char. 45[0]; Figure 3). Craniodistal edge of metacarpal III extends further distally than caudodistal edge (§char. 48[0]; Figure 6).

*Eurylaimides*: Carpometacarpus is straight dorsoventrally (char. 6[0]; Figure 2), occurs independently multiple times throughout Eupasseres. A distally positioned cranial process in relation to the alular process (char. 10[0]; Figure 4); this character does not optimise as a synapomorphy; however, it is notable due to its occurrence in fossil passerines. Character state 10(0) occurs in most sampled taxa within Eurylaimides, occurs a few times independently within Tyrannida, and is variable across Passeri. Distal margin of alular process in line with fovea ligamentalis ventralis (§char. 18[1]; Figure 5), and fovea lig. ventralis distocaudal to proximal edge of pisiform process (§char. 20[0]; Figure 6). Sulcus for M. flexor digiti III terminates distally of pisiform process (§char. 21[0]; Figure 5). Shallow sulcus for M. flexor digiti III (§char. 22[0]; Figure 2). Metacarpal III is relatively straight (§char. 26[0]; Figure 5). Multiple protrusions present along dorsal edge of metacarpal III (§char. 32[1]; Figure 4). Cranial margin of metacarpal II is irregular, not straight (§char. 34[1]; Figure 4) and dentiform process is absent (§char. 35[0]; Figure 4). Sulcus tendineus is shallow (§char. 39[0]; Figure 3), and the ridge along the cranial edge of the sulcus is incomplete (§char. 42[0]; Figure 3). Intersulcus bridge forming distinctive hole at distal sulcus tendineus absent (§char. 44[0]; Figure 3). Metacarpal III projects slightly distally beyond metacarpal II (char. 46[1], also present in Tyrannides, *Zygodactylus luberonensis* and Piciformes). Facies articularis digiti III obscured by distal tip of metacarpal III (char. 47[1]; Figure 5), also present in Tyrannides and independently within Passeri, Acanthisitti and some non‐passeriforms. Characters 6, 46 and 47 did not optimise as synapomorphies due to the same state being inferred for the ancestral node of Tyrannida, resulting in ambiguity at the Tyranni node.

*Tyrannides*: No additional character combinations were recovered for this clade.

*Furnariida*: Fovea lig. ventralis distocaudal to proximal edge of pisiform process (§char. 20[0]). Sulcus tendineus is shallow (§char. 39[0]), and the ridge along the cranial edge of sulcus tendineus is incomplete (§char. 42[0]). However, *Glyphorynchus spirurus* has independently acquired char. 39(1). Metacarpal III projects substantially distally beyond metacarpal II (char. 46[2]). This character is reversed to 46(1) in *Formicarius analis* and *Furnarius rufus*; however, Furnariida is the only Tyranni subclade in our sample that exhibits 46(2). Character 46(2) did not optimise as a synapomorphy due to (2) occurring at the Acanthisitti and Passeri nodes resulting in ambiguity at the Tyranni and Tyrannides nodes. Additionally, Furnariida is the only passeriform clade in our sample with representatives exhibiting a distinctive hole at the distal end of the sulcus tendineus (char. 44[1]), as seen in Dendrocolaptidae, Scleruridae and *Xenops minutus*.

*Tyrannida*: Cranial margin of fovea carpalis caudalis is continuous with caudoproximal margin of intermetacarpal process (§char. 13[0]; Figure 3), widely present across total‐clade Passeriformes. Sulcus for M. flexor digiti III terminates distally of pisiform process (§char. 21[0]). Distinct protrusions along ventral edge of metacarpal III are present so edge is clearly undulating (char. 27[1]; Figure 6); this did not optimise as a synapomorphy due to 27(1) being present across the sister clade, resulting in ambiguity at the Tyrannides node. Cranial margin of caudodistal region of metacarpal II is moderately expanded cranially (§char. 37[1]; Figure 4), present in several non‐passeriform lineages and is ancestral for total‐clade Passeriformes. Sulcus tendineus is moderately deep (char. 39[1]); this character is reversed in *Rupicola peruvianus* and occurs in one other Tyranni taxon independently (*Glyphorynchus spirurus*). Ridge along cranial margin of distal sulcus tendineus is clearly continuous (char. 42[1]); this character is reversed in *Tityra semifasciata* but 42(1) does not occur elsewhere within the Tyranni taxa sampled, although it has independently arisen in Acanthisitti and several Passeri lineages. Intersulcus bridge forming distinctive hole at distal sulcus tendineus absent (§char. 44[0]). Metacarpal III projects slightly distally beyond metacarpal II (char. 46[1]); this did not optimise as a synapomorphy due to 46(1) also occurring at the Eurylaimides node, resulting in ambiguity at the Tyrannides node.

*Passeri*: Distal margin of fovea carpalis caudalis is clearly delimited by a ridge, separating it from the proximal intermetacarpal space (§char. 11[1]; Figure 3), also present in several lineages within Tyranni in addition to non‐passeriforms. Dorsal surface of caudodistal region of metacarpal II is planar (*char. 38[1]); within total‐clade Passeriformes, this character is unique to Passeri but also occurs in *Capito niger* and *Cariama cristata*. Intersulcus bridge forming distinctive hole at distal sulcus tendineus absent (§char. 44[0]). Metacarpal III projects substantially distally beyond metacarpal II (char. 46[2]), present in Furnariida, Acanthisitti and independently in *Merops orientalis*. Character 46(2) did not optimise as a synapomorphy due to 46(2) occurring at the Acanthisitti and Furnariida nodes, resulting in ambiguity at the crown Passeriformes and Eupasseres nodes. Distal ventral fossa present (char. 51[1]; Figure 6). This character is present in almost all sampled extant Passeri taxa; however, it is absent in *Atrichornis rufescens*, *Dasyornis broadbenti* and *Pnoepyga pusilla*. Character 51(1) is considered in this list despite these exceptions because all three taxa are highly specialised for ground‐dwelling lifestyles, with short, dorsoventrally and craniocaudally curved carpometacarpi—likely due to convergence related to a reluctance to fly (Boles, 1995). Character 51(1) also arises multiple times independently within Tyranni.

*Corvides + Passerides*: No additional character combinations were recovered for this clade.

*Corvides*: Trochlea carpalis is oblong in shape (§char. 16[0]). Sulcus for M. flexor digiti III proximally terminates approximately in line with pisiform process (§char. 21[1]). Although not ubiquitously present across Corvides, a completely straight, uniform cranial margin of metacarpal II (char. 34[0]) is not present in any other Passeri lineage; 34(0) is also present within Tyranni and several non‐passeriform lineages. Distal ventral fossa present (§char. 51[1]).

*Passerides*: Cranial margin of metacarpal II is irregular, not straight (§char. 34[1]) and dentiform process present (§char. 35[1]), also present in other Passeri subclades, Tyranni and non‐passeriforms.

### 3.2 Bayesian phylogenetic analyses

Analysis 1, conducted under a complete molecular scaffold with the position of *Zygodactylus luberonensis* constrained as a stem passeriform, recovered all fossils that were previously suggested to show suboscine affinities (SMNS 59466/1, NT‐LBR‐014, *W. weissi* SMNK‐PAL 3980, SMF Av 504; Table 1; Figure S1) within crown Tyrannida with reasonably strong support (0.79 BPP), within crown Tyrannides with slightly stronger support (0.87 BPP), and within crown Tyranni with very strong support (0.97 BPP). All fossils that were previously suggested to be oscines (SMNS 59466/3–6, SMF Av 514; Table 1), as well as the fossils with unknown passeriform affinities (SMNS 59466/14 & SMF Av 517; Table 1), were recovered within the clade uniting Climacterides and its sister clade with reasonably strong support (0.87 BPP), and were recovered within crown Passeri with very strong support (0.98 BPP).

Analysis 2 included SMNS 59466/3 and excluded all other non‐suboscine fossils (Figure S2). SMNS 59466/3 was recovered in a polytomy with Corvides and Passerides with weak support (0.59 BPP), and within the clade uniting Climacterides and its sister clade with strong support (0.95 BPP). Analysis 3 included SMNS 59466/4 (Figure S3) which was recovered in a polytomy with Sylviida and Muscicapida + Passerida with weak support (0.54 BPP). SMNS 59466/4 was recovered within Corvides + Passerides with reasonably strong support (0.886 BPP), and within the clade uniting Meliphagides and its sister clade with strong support (0.92 BPP). Analysis four included SMNS 59466/5 (Figure S4) which was recovered in a polytomy with Callaeida and Petroicida + (Sylviida + (Muscicapida + Passerida)) with reasonably strong support (0.78 BPP). SMNS 59466/5 was recovered within Corvides + Passerides with strong support (0.93 BPP). The phylogenetic placement of all fossils investigated here are summarised in Figure 8.

## 4 DISCUSSION

This study represents the most comprehensive exploration of passerine carpometacarpal morphology to date, with an unprecedentedly diverse taxon sample exceeding that of any previous morphological investigation of passerines. We identified diagnostic combinations of carpometacarpal character states for major passerine subclades, and our phylogenetic analyses recovered fossil passerine carpometacarpi from the Oligocene of Europe as early representatives of major subclades within crown Passeriformes.

Our detailed evaluation of carpometacarpal characters reveals substantial homoplasy in the passerine carpometacarpus, and highlights the need to re‐evaluate previously described anatomical characters and incorporate both phylogenetic analyses and character state optimisation to develop informative lists of diagnostic character combinations for key passerine clades. We believe this approach will be necessary for robustly placing fragmentary passerine fossils into an evolutionary context. Nonetheless, these results have implications for re‐calibrating the timeline of passerine evolutionary history, and bear on the complex biogeographic origins of one of the deepest passerine subclades, the suboscines (Tyranni).

### 4.1 Phylogenetic affinities of Oligocene suboscine fossils from Europe

We analysed several controversial crown passerine fossils from the Oligocene of Europe (Figure 7), which have considerable potential to illuminate aspects of early passeriform evolution. Most of these fossils were incorporated into phylogenetic analyses for the first time here. Our phylogenetic results recovered four fossil specimens (SMF Av 504; NT‐LBR‐014; SMNK‐PAL 3980; SMNS 59466/1) as representatives of crown Tyranni across all analyses (crown‐group suboscines; Figure 8). These fossils were previously attributed to total‐clade Tyranni, but the basis for our assignment differs from prior work (Table 1). Earlier studies suggested that these fossils exhibited diagnostic character states for Tyranni and lacked diagnostic features of Passeri (Manegold, 2008b; Mayr & Manegold, 2006a, 2006b). From our character optimisation results, we find that char. 38(0) (a protruding dorsal surface of the caudodistal region of metacarpal II; Figure 3; Manegold, 2008b) is ubiquitously present across Tyranni, although this character state is plesiomorphic for crown Passeriformes. We find that the alternate state (38[1]: a planar dorsal surface of the caudodistal region of metacarpal II) is diagnostic for crown Passeri, although it also occurs independently in the non‐passeriform barbet *Capito niger* and seriema *Cariama cristata* and so cannot be considered a unique synapomorphy. Other character states previously proposed as diagnostic of Tyranni, such as char. 49(1) (presence of a craniodistal process; Figure 6; Mourer‐Chauviré et al., 1989) are not only absent in several extant Tyranni taxa (e.g., *Melanopareia torquata*, *Thamnophilus torquata*), but also present in several major Passeri subclades, including Corvides, Petroicida, Sylviida and Muscicapida, and therefore cannot be considered diagnostic for crown Tyranni. We recovered char. 40(0) (distal end of sulcus tendineus does not broaden into a fossa) as the only unreversed carpometacarpal synapomorphy of crown Tyranni; however, this character state arises multiple times independently across crown Passeri, exemplifying the considerable degree of homoplasy in the carpometacarpi of Passeriformes.

In this study, we split the original character referring to the craniodistal process (Mourer‐Chauviré et al., 1989) into two, where char. 49(1) (presence of a craniodistal process) is conditional on char. 48(0) (craniodistal edge of metacarpal III extends further distally than the caudodistal edge to produce an oblique distal end to metacarpal III; Figure 6). Character state 48(0) is ubiquitously present across Tyranni but is also widely present across crown Passeriformes. Character state 51(0) (absence of a distal ventral fossa; Figure 6)—also previously considered diagnostic for Tyranni (Manegold, 2008b)—is in fact highly variable across the clade, with state 51(1) arising multiple times independently within Tyranni.

All of the early Oligocene fossils investigated here (SMNK‐PAL 3980; NT‐LBR‐014; SMF Av 504; Figure 7a–c) share several phylogenetically informative character states. Character state 46(1) (metacarpal III only extending slightly distally beyond metacarpal II; Figure 5) supports the exclusion of the early Oligocene fossils from crown Furnariida, as most furnariidans exhibit char. 46(2) (metacarpal III extending substantially distally beyond metacarpal II; although this condition is reversed in two extant taxa—*Formicarius analis* and *Furnarius rufus*—within our sample). Notably, in its original description, *W. weissi* (SMNK‐PAL 3980; Figure 7a) was said to exhibit a metacarpal III that does not extend distally beyond metacarpal II as seen in all other crown passeriforms (Mayr & Manegold, 2006b). However, we argue that the caudodistal portion of metacarpal III is in the same position as seen in most extant suboscine taxa, such as tyrannidans and eurylaimidans, and that its more ventrally oriented craniodistal portion may be hidden, which may also obscure whether the craniodistal portion of metacarpal III extends further distally than the caudodistal portion (char. 48[0]), as well as the presence of a craniodistal process (char. 49[1]). In the future, it may be possible to evaluate the presence or absence of these features in *W. weissi* with non‐invasive μCT scanning, which would shed new light on the phylogenetic affinities of this critical specimen.

A moderately deep sulcus tendineus (char. 39[1]; Figure 3) and a clearly continuous ridge along the cranial margin of the sulcus tendineus (char. 42[1]; Figure 3) support the exclusion of all the early Oligocene fossils from crown Furnariida and crown Eurylaimides. By contrast, both of these characters are present in most extant representatives of Tyrannida. Character state 39(1) is present in all tyrannidans sampled except *Rupicola peruvianus* and is present in only one non‐tyrannidan suboscine (*Glyphorynchus spirurus*). This character state is extremely variable in non‐suboscine passeriforms. Within Tyranni, char. 42(1) appears to be unique to Tyrannida, and only one tyrannidan in our sample did not exhibit this state (*Tityra semifasciata*). Character 42(1) also arises independently in some Passeri lineages as well as Acanthisitti, but was not recovered as the plesiomorphic condition for crown Passeriformes. However, all three early Oligocene fossils differ from extant tyrannidans in that the cranial margin of the craniodistal region of metacarpal II is approximately level or only slightly expanded cranially (char. 37[0]; Figure 4), as opposed to being moderately expanded cranially (37[1]). Additionally, *W*. *weissi* exhibits a cranial margin of fovea carpalis caudalis that is not continuous with the caudoproximal margin of the intermetacarpal process (char. 13[0]; Figure 3).

A distally positioned cranial process in relation to the alular process (char. 10[0]; Figure 4) is observed in all the Oligocene suboscine fossils examined (Figure 7b). Fjeldså et al. (2020) interpreted this character as present within Eurylaimidae and used it to justify the placement of *W. weissi* as a crown eurylaimid in a recent qualitative evaluation of the fossil. However, our results suggest that this character state is plesiomorphic for the broader clade Eurylaimides; as such, it cannot be used to diagnose the fossil as a crown eurylaimid.

By contrast, char. 10(0) is inferred to have arisen independently several times across a limited number of extant tyrannidans, and is absent in Furnariida. The ancestral state for char. 10 is ambiguous for crown Tyranni, but for Tyrannides, it was inferred to be 10(1). In light of our phylogenetic analyses and the diagnostic character combinations we infer for this clade, it would be reasonable to propose that the early Oligocene fossils are either representatives of stem‐group Tyrannides (supported by the fact that they exhibit a potentially plesiomorphic state for char. 10), or representatives of stem‐group Tyrannida (supported by the fact that they exhibit two character states that are unique (42[1]) or almost unique (39[1]) to Tyrannida within the wider context of Tyranni). The latter scenario aligns with the description of NT‐LBR‐014 that suggested a stem tyrannidan placement for this fossil, based on anatomical evidence from across the skeleton (Riamon et al., 2020). We cannot conclusively rule out a position for these fossils within total‐clade Eurylaimides; however, as there is no strong support for an affinity with this clade, it seems most plausible that the early Oligocene fossils fall outside crown Eurylaimides, with their affinities lying elsewhere within crown Tyranni. For early Oligocene fossil passerines exhibiting additional skeletal material beyond the carpometacarpus, further interpretation of the skeleton with comprehensively evaluated morphological characters will be necessary to gain a more complete understanding of their phylogenetic placement.

### 4.2 Phylogenetic affinities of Oligocene oscine fossils from Europe

The remaining crown passerine fossils investigated here were recovered within crown Passeri with strong support (Figure 8), including two carpometacarpi (SMNS 59466/14 & SMF Av 517; Figure 7j,k) that were assigned to Passeriformes *incertae sedis* (Manegold, 2008b). These two carpometacarpi were originally unassigned to a more specific phylogenetic placement within Passeriformes because they exhibit an unfused intermetacarpal process (char. 29[0]; Figure 4). An intermetacarpal process fused to metacarpal III has long been considered a synapomorphy of crown Passeriformes (Mayr & Manegold, 2006b), and all extant passerines in our sample exhibited this character state (29[1]). It is possible that the unfused intermetacarpal process on these fossils is indicative of a skeletally immature ontogenetic state (Manegold, 2008b), although this remains challenging to assess.

Manegold (2008b) described two diagnostic carpometacarpal character states for crown Passeri: a planar dorsal surface of the caudodistal region of metacarpal II (char. 38[1]; Figure 3), and the presence of a distal ventral fossa (char. 51[1]; Figure 6). Character state 38(1) is almost unique to, and ubiquitously present within, Passeri, only otherwise occurring in the piciform barbet *Capito niger* within our dataset. As discussed above, char. (51[1]) also occurs independently across Tyranni. Character state 38(1) is observable in all the oscine fossils examined (Figure 7e–i) except SMNS 59466/14 and SMF Av 517 (Figure 7j,k) in which the character is unobservable due to breakage, and 51(1) is observable in half the fossil carpometacarpi (SMNS 59466/3, SMNS 59466/5 and SMNS 59466/6, as well as suboscine fossil NT‐LBR‐014; Figure 7b,e,g,h). From what is observable, all the oscine fossils investigated appear to exhibit character states that are ubiquitously present in all extant Passeri (except the aforementioned unfused intermetacarpal process). However, it is more difficult to assign them to, or exclude them from, subclades within crown Passeri, based on the character state combinations they exhibit.

All the oscine fossils exhibit a dentiform process (char. 35[1]; Figure 5), which may exclude them from the Corvides subclade Malaconotoidea + Corvoidea, in which this feature is ubiquitously absent. Another notable character, an obliquely truncated metacarpal III (char. 48[0]; Figure 6), was first mentioned by Manegold (2008b) and used by Fjeldså et al. (2020) to justify the hypothesis that all the oscine fossils except SMF Av 514 (in which it is square shaped; char. 48[1]; Figure 6), were early representatives of Passerides. From our widespread sampling across Passeri, we find that char. 48(0) arose multiple times independently, suggesting that an affinity with Passerides may not be justified. This character state is common in several subclades within Passeri, such as the sequential sister taxa to the Passerides + Corvides clade, Corvides, the sequential sister taxa to the Sylviida + (Muscicapida + Passerida) clade, and within Muscicapida and Sylviida. Indeed, almost all taxa sampled within Sylviida exhibited this character state. Therefore, we cannot use this character to support the presence of early representatives of Passerides in the late Oligocene of Europe. For now, these oscine carpometacarpi fossils may not be assignable to any particular subclade beyond what was recovered in our phylogenetic analyses (Figure 8). The carpometacarpus may exhibit even more homoplasy across Passeri than Tyranni, exemplified by poor phylogenetic resolution within Passeri (see Figures S5 and S6), rendering taxonomic assignment of these fossils particularly challenging.

### 4.3 Implications for the evolutionary history of crown Passeriformes

We have shown that the passerine carpometacarpus is characterised by widespread homoplasy, epitomised by the lack of truly unique synapomorphies diagnosing passerine subclades. Nevertheless, our detailed evaluation of morphological variation across a broad taxon sample identified various phylogenetically‐informative traits. These place constraints on interpretations of the phylogenetic placement of passerine fossils from the Oligocene of Europe. Our phylogenetic analyses corroborate prior hypotheses that representatives of crown Tyranni were present in Europe from the early Oligocene (e.g., Riamon et al., 2020), and co‐occurred with representatives of crown Passeri during the late Oligocene (Manegold, 2008b). These results are consistent with the hypothesis that representatives of crown Passeri dispersed out of Australasia by the end of the Oligocene (Moyle et al., 2016; Oliveros et al., 2019).

The oscine fossils sampled in this study are more morphologically disparate than the suboscine fossils studied (Figure 7), and were recovered in several different positions across the Passeri crown group (Figure 8). By contrast, early Oligocene suboscine carpometacarpi were generally morphologically similar and were recovered as one another's respective sister taxa (Supporting Information), while the late Oligocene suboscine fossil examined here diverged somewhat in its morphology. All the suboscine fossils sampled most likely derive from different species based on size differences (Manegold, 2008b; Mayr & Manegold, 2006a, 2006b; Riamon et al., 2020). Amongst the early Oligocene suboscine fossils, our phylogenetic analyses indicate that *Wieslochia weissi* is neither a stem‐group eupasseran (Mayr & Manegold, 2006b) nor a crown eurylaimid (Fjeldså et al., 2020) as previously hypothesised, but likely resides somewhere within crown Tyranni, as opposed to falling within its stem group (Ksepka et al., 2019). Further investigation across the skeleton of articulated specimens will be necessary to clarify their phylogenetic placement within total‐clade Tyranni. From our analyses and evaluation of diagnostic character states, it seems plausible that the early Oligocene suboscine specimens investigated here are closely related to one‐another and potentially represent stem‐group members of either Tyrannides or Tyrannida.

Delineating between plesiomorphic and apomorphic character states has frequently proven challenging due to high rates of homoplasy across the passerine carpometacarpus, complicating the assessment of the phylogenetic position of fossils. The work presented here has exemplified the necessity to study passerine comparative morphology in a broadly‐sampled phylogenetic context and has demonstrated that at least one character‐rich element of the skeleton—the carpometacarpus—exhibits significant morphological lability. Characterising additional elements of the passerine skeleton at a comparable level of detail will be critical for furthering our understanding of passerine morphological evolution, and clarifying key patterns from the earliest stages of passerine evolutionary history.

## AUTHOR CONTRIBUTIONS

EMS and DJF conceptualised the project. All authors contributed to data collection. EMS and JMTN compiled the character list. EMS conducted the analyses, made the figures and wrote the first draft. All authors reviewed and edited the final manuscript. DJF, RBJB and JMTN supervised the project.

### OPEN RESEARCH BADGES

This article has earned Open Data badge. Data and materials are available at www.morphosource.org/projects/000454063/about?locale=en.

## Supporting information


Figures S1‐6

Appendix S1

Table S1

Table S2

Appendix S2

## Data Availability

All 3D models are available from Morphosource and links to individual media can be found in Table S1. All other datafiles are provided in the Supporting Information.
