# Supplementary material for: Comparative anatomy of the passerine carpometacarpus helps illuminate the early fossil record of crown Passeriformes
Source: J Anat. 2022 Sep 7;242(3):495–509. doi: 10.1111/joa.13761 (PMC9919509; doi:10.1111/joa.13761)
Supplement: Supplementary file 1 — Figures S1‐6 [file JOA-242-495-s005.zip › joa13761-sup-0001-Supinfo1.docx]

**Supporting Information for “Comparative anatomy of the passerine carpometacarpus helps illuminate the early fossil record of crown Passeriformes”**

Table of Contents

[1. Table S1: List of Specimens 1](#_Toc101280160)

[2. Table S2: List of Characters 1](#_Toc101280161)

[3. Character optimisation analysis 2](#_Toc101280162)

[i. TNT script 2](#_Toc101280163)

[ii. Results: Mapped synapomorphies 2](#_Toc101280164)

[iii. Results: Mapped character state transitions 2](#_Toc101280165)

[4. Diagnostic character combinations for additional passerine subclades 2](#_Toc101280166)

[5. Bayesian phylogenetic analyses 1-4 5](#_Toc101280167)

[i. Scripts 5](#_Toc101280168)

[ii. Trees for analyses 1-4 5](#_Toc101280169)

[6. Additional Bayesian phylogenetic analyses 6](#_Toc101280170)

[i. Methods 6](#_Toc101280171)

[ii. Scripts 7](#_Toc101280172)

[iii. Results 7](#_Toc101280173)

[iv. Trees for analyses S1 and S2 8](#_Toc101280174)

# **Table S1: List of Specimens**

See ‘Table_S1_SuppInfo.xlsx’.

# **Table S2: List of Characters**

See ‘Table_S2_SuppInfo.xlsx.

# **Character optimisation analysis**

## *TNT script*

See ‘TNT_SuppInfo.tnt’.

## *Results: Mapped synapomorphies*

See ‘synaps_SuppInfo.txt’.

## *Results: Mapped character state transitions*

See ‘mapped_states_SuppInfo.txt’.

# **Diagnostic character combinations for additional passerine subclades**

Acanthisitti: Carpometacarpus slightly bowed dorsoventrally (char. 6(1); Fig. 2), also present in several non-passeriforms in our sample (Trogon melanurus, Capito niger, Picus viridis) as well as within Furnariida and Passeri subclades. Distal position of cranial process in relation to approximate midpoint of intermetacarpal process (char. 9(0); Fig. 3), also present in Zygodactylus luberonensis. Distal margin of fovea carpalis caudalis is continuous with proximal intermetacarpal space (*char. 11(0); Fig. 3), also arises independently multiple times in Tyranni. Cranial margin of fovea carpalis caudalis continuous with caudoproximal margin of intermetacarpal process (§char. 13(0); Fig. 3). Shallow fovea carpalis caudalis (§char. 14(0); Fig. 4). Fossa infratrochlearis is deeply recessed proximal to pisiform process (*char. 15(1); Fig. 5), widely present and variable across crown Passeriformes and non-passerines in our sample. Trochlea carpalis ventralis is oblong in shape (§char. 16(0); Fig. 6). Distal margin of metacarpal I is distal relative to fovea ligamentalis ventralis (§char. 18(0); Fig. 5). Deep fovea ligamentalis ventralis (char. 19(1); Fig. 5), independently evolved in many eupasseran lineages and Picus viridis. Fovea ligamentalis ventralis distocaudal to proximal edge of pisiform process (§char. 20(0); Fig. 6). Sulcus for M. flexor digiti III terminates distally of pisiform process (§char. 21(0); Fig. 5). Shallow sulcus for M. flexor digiti III (§char. 22(0); Fig. 2) and proximal margin barely or not distinguishable (§char. 23(0); Fig. 5). Metacarpal III is relatively straight (§char. 26(0); Fig. 5). Caudal margin of intermetacarpal process does not protrude caudally by a substantial amount (*char. 30(0); Fig. 4). Multiple protrusions present along dorsal edge of metacarpal III (§char. 32(1); Fig. 4). Intermetacarpal process perfectly superimposes proximal protrusion of metacarpal III (*char. 33(0); Fig. 4). Cranial margin of metacarpal II is irregular, not straight (§char. 34(1); Fig. 4). Dentiform process absent (char. 35(0); Fig. 4), absent in many non-passeriforms as well as within Tyranni and Corvides. Cranial margin of craniodistal region of metacarpal II is approximately level to proximal region (*char. 37(0); Fig. 4), also present in Tyranni. Dorsal surface of caudodistal region of metacarpal II protrudes dorsally (§char. 38(0); Fig. 3). Distal sulcus tendineus broadens into a fossa (§char. 40(1); Fig. 3). Continuous ridge along cranial margin of sulcus tendineus present (*char. 42(1); Fig. 3), also present in Tyrannida, and ridge is approximately the same width along its length (§char. 43(0); Fig. 3). Intersulcus bridge forming distinctive hole at distal sulcus tendineus absent (§char. 44(0); Fig. 3). Distinct process on facies articularis digiti II absent (§char. 45(0); Fig. 3). Metacarpal III projects substantially beyond metacarpal II (char. 46(2)), also present in Furnariida and Passeri. Craniodistal edge of distal metacarpal III extends further distally than caudodistal edge (char. 48(0); Fig. 6). Distal ventral fossa absent (§char. 51(0); Fig. 6).

Meliphagides: Sulcus for M. flexor digiti III proximally terminates approximately in line with pisiform process (§char. 21(1)), also occurs in Furnariida and several non-passeriform taxa. Multiple protrusions present along dorsal edge of metacarpal III (§char. 32(1)). Cranial margin of metacarpal II is irregular, not straight (§char. 34(1)) and dentiform process present (§char. 35(1)). Cranial margin of caudodistal region of metacarpal II is greatly expanded cranially (§char. 37(2)). Sulcus tendineus moderately deep (*char. 39(1)), also occurs in Acanthisitti, Tyrannida, Passeri and several coraciimorphs. Distinct process on facies articularis digiti II absent (§char. 45(0)). Facies articularis digiti III obscured by distal tip of metacarpal III (§char. 47(0)).

Sylviida: Trochlea carpalis ventralis oblong in shape (§char. 16(0)). Sulcus for M. flexor digiti III proximally terminates approximately in line with pisiform process (§char. 21(1)). Multiple protrusions present along dorsal edge of metacarpal III (§char. 32(1)).

Muscicapida + Passerida: No additional character combinations were recovered for this clade.

Muscicapida: Carpometacarpus slightly bowed (§char. 6(1)). Trochlea carpalis ventralis oblong in shape (§char. 16(0)). Although not ubiquitously present across Muscicapida, the sulcus for M. flexor digiti III proximally terminating distal to the pisiform process (char. 21(0)) does not occur in any other Passerides lineage; 21(0) is also present within Climacterides, Tyranni, Acanthisitti and several non-passeriform lineages. Intermetacarpal process does not protrude caudally beyond proximal edge of metacarpal III (§char. 30(0)), also occurs across other Passeri subclades, Tyranni and Acanthisitti. Multiple protrusions present along dorsal edge of metacarpal III (§char. 32(1)). Dentiform process arises distally of proximodistal midpoint along metacarpal II (§char. 36(1); Fig. 4), also arises in Sylviida, Petroicida, Callaeida, Tyranni, Pardalotus striatus and Zygodactylus luberonensis. Craniocaudal width of ridge along cranial margin of distal sulcus tendineus is uniform along length of ridge (§char. 43(0)), arises multiple times independently across crown Passeriformes and is present in non-passerine lineages.

Passerida: Sulcus for M. flexor digiti III proximally terminates approximately in line with pisiform process (§char. 21(1)). Distinct protrusions on the ventral edge of metacarpal III are absent (§char. 27(0)). Distinct process on facies articularis digiti II absent (§char. 45(0)). Two distinct processes on the distal edge of metacarpal III are absent (§char. 50(0); Fig. 6).

# **Bayesian phylogenetic analyses 1-4**

## *Scripts*

For nexus files for each main Bayesian analysis, see ‘Analysis1_SuppInfo.nex’, ‘Analysis2_SuppInfo.nex’, ‘Analysis3_SuppInfo.nex’ and ‘Analysis4_SuppInfo.nex’. For the complete topological constraint, see ‘complete_constraint_SuppInfo.phy’. For the R script to generate the topological constraint code for MrBayes, see ‘paleotree_constraints_SuppInfo.R’.

## *Trees for analyses 1-4*

Bayesian phylogenetic results for analyses 1-4 are presented in the file ‘SuppInfo_Figures.pdf’. All analyses are summarised as 50% majority rule trees. Node labels represent Bayesian posterior probabilities (BPP). Fossils are highlighted in blue and the node for the clade in which they are illustrated within in Figure 7 is highlighted in red (only nodes exceeding 0.90 BPP).

Figure S1: Results from Bayesian phylogenetic Analysis 1 including all crown passeriform fossils in Table 1 as unconstrained. See ‘Analysis_1_SuppInfo.tre’ for the full tree.

Figure S2: Results from Bayesian phylogenetic Analysis 2 including all suboscine fossils, but excluding all non-suboscine fossils except SMNS 59466/3, as unconstrained. See ‘Analysis_2_SuppInfo.tre’ for the full tree.

Figure S3: Results from Bayesian phylogenetic Analysis 3 including all suboscine fossils, but excluding all non-suboscine fossils except SMNS 59466/4, as unconstrained. See ‘Analysis_3_SuppInfo.tre’ for the full tree.

Figure S4: Results from Bayesian phylogenetic Analysis 4 including all suboscine fossils, but excluding all non-suboscine fossils except SMNS 59466/5, as unconstrained. See ‘Analysis_4_SuppInfo.tre’ for the full tree.

# **Additional Bayesian phylogenetic analyses**

## *Methods*

We conducted two additional Bayesian phylogenetic analyses (S1 & S2) that were carried out in MrBayes V3.2.2 (Ronquist et al., 2012) to evaluate whether inferences from our morphological carpometacarpus dataset could replicate the phylogenetic relationships recovered from the Oliveros et al. (2019) and Harvey et al. (2020) molecular datasets. We excluded characters 1-5 which are continuous and therefore could not be included. These analyses were performed for two runs with four chains and 30 million replicates sampled every 4,000 generations with a chain temperature of 0.1 and burn-in set to 0.25. The likelihood model priors were sampled from a gamma distribution with four rate categories with variable coding for morphological data. Analysis S1 was summarised as a 50% majority rule tree (contype = Halfcompat). Analysis S2 was summarised with all recovered nodes presented (contype = Allcompat). A partial topological constraint was applied on a subset of extant taxa for both analyses. Constrained nodes were resolved by Oliveros et al. (2019) and Harvey et al. (2020) and included the following taxa: Columba livia; Trogon melanurus; Merops orientalis; Picus viridis; Cariama cristata; Falco sparverius; Nestor notabilis; Acanthisitta chloris; Eurylaimus ochromalus; Thamnophilus torquatus; Tyrannus tyrannus; Menura novaehollandiae; Corvus brachyrhynchos; Poecile atricapillus and Emberiza calandra. Zygodactylus luberonensis SMF Av519 was left unconstrained. No other fossil taxa were included.

## *Scripts*

For nexus files for both additional Bayesian analyses, see ‘Analysis_S1_SuppInfo.nex’ and ‘Analysis_S2_SuppInfo.nex’. For the partial topological constraint, see ‘partial_constraint_SuppInfo.phy’.

## *Results*

Analysis S1 (Fig. S5), conducted under a partial topological constraint (see above) and summarised as a 50% majority rule tree, resolved total-clade Passeriformes as a monophyletic group with strong support (0.99 BPP), although internal relationships were largely unresolved. Passeri was resolved as a clade (0.88 BPP), although all main subclades within Passeri collapsed to a polytomy. Nonetheless, some non-oscine taxa resolved within the Passeri polytomy; the furnariidan taxa Melanopareia torquata and Liosceles thoracicus resolved as sister taxa within Passeri, in addition to the piciform Capito niger. Acanthisitti formed a monophyletic group (0.90 BPP), and Zygodactylus luberonensis, Acanthisitti, Passeri, and all other suboscines formed a polytomy. Analysis S2 (Fig. S6) was run under the same parameters as S1 but summarised with all nodes presented on the tree, therefore includes clades with <0.50 BPP support. Overall, Tyranni is better supported than Passeri in terms of related taxa clustering together. Passeri resolves as a clade, although see above for additional taxa that resolved within Passeri; however, taxa generally cluster more randomly as opposed to being in related groups, potentially as a consequence of homoplasy.

## *Trees for analyses S1 and S2*

Bayesian phylogenetic results for analyses S1 and S2 are presented in the file ‘Figures_S1-S6_SuppInfo.pdf’. Both analyses were conducted with a partial topological constraint (see above). Node labels represent Bayesian posterior probabilities (BPP).

Figure S5: Results from Bayesian phylogenetic Analysis S1 summarised as a 50% majority rule tree. See ‘Analysis_S1_SuppInfo.tre’ for the full tree.

Figure S6: Results from Bayesian phylogenetic Analysis S2 summarised as a consensus tree with all nodes presented. See ‘Analysis_S2_SuppInfo.tre’ for the full tree.
