# Supplementary figures and images for: Comparative anatomy of the passerine carpometacarpus helps illuminate the early fossil record of crown Passeriformes
Source: J Anat. 2022 Sep 7;242(3):495–509. doi: 10.1111/joa.13761 (PMC9919509; doi:10.1111/joa.13761)

Figure S1

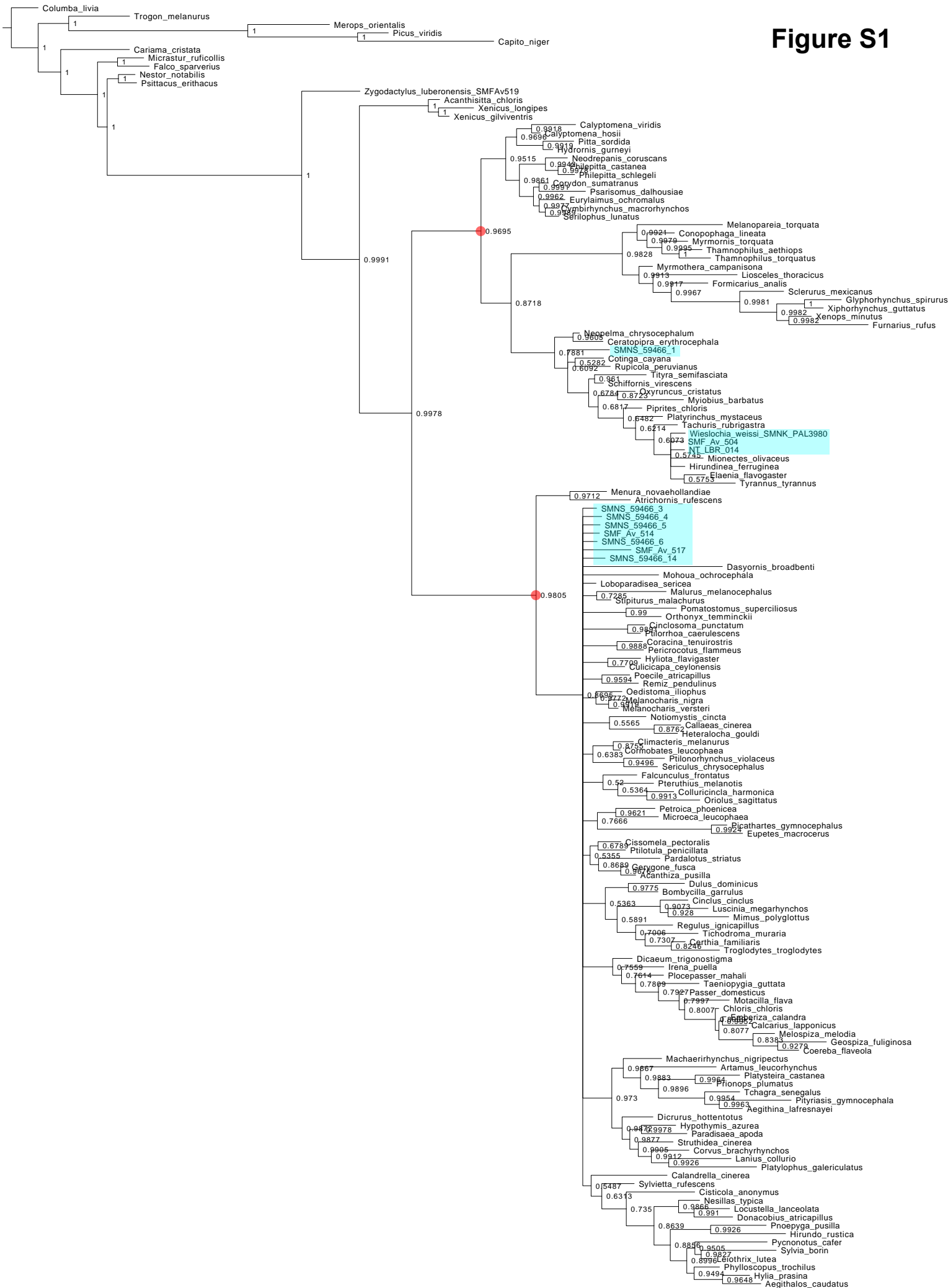

Figure S2

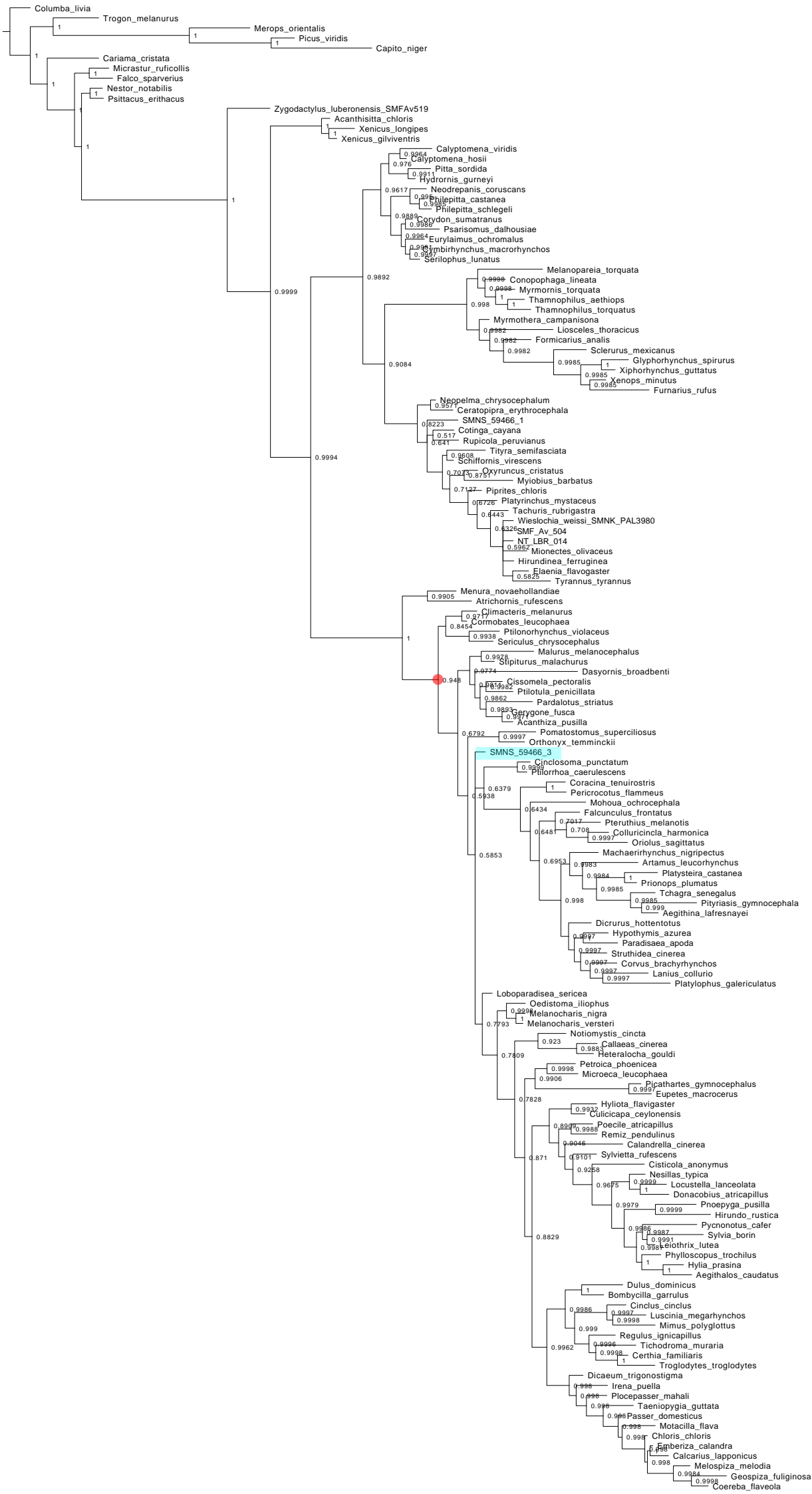

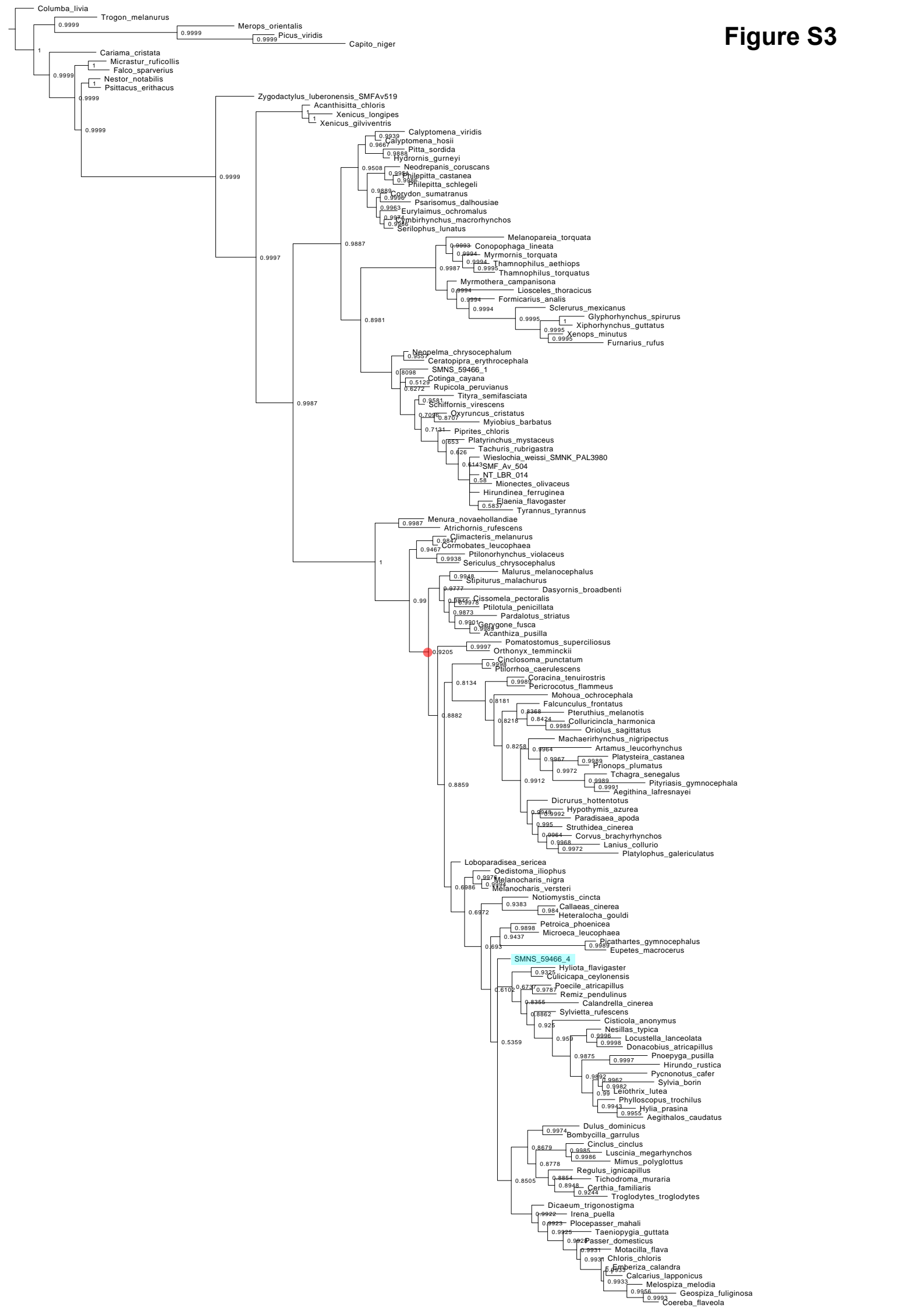

Figure S4

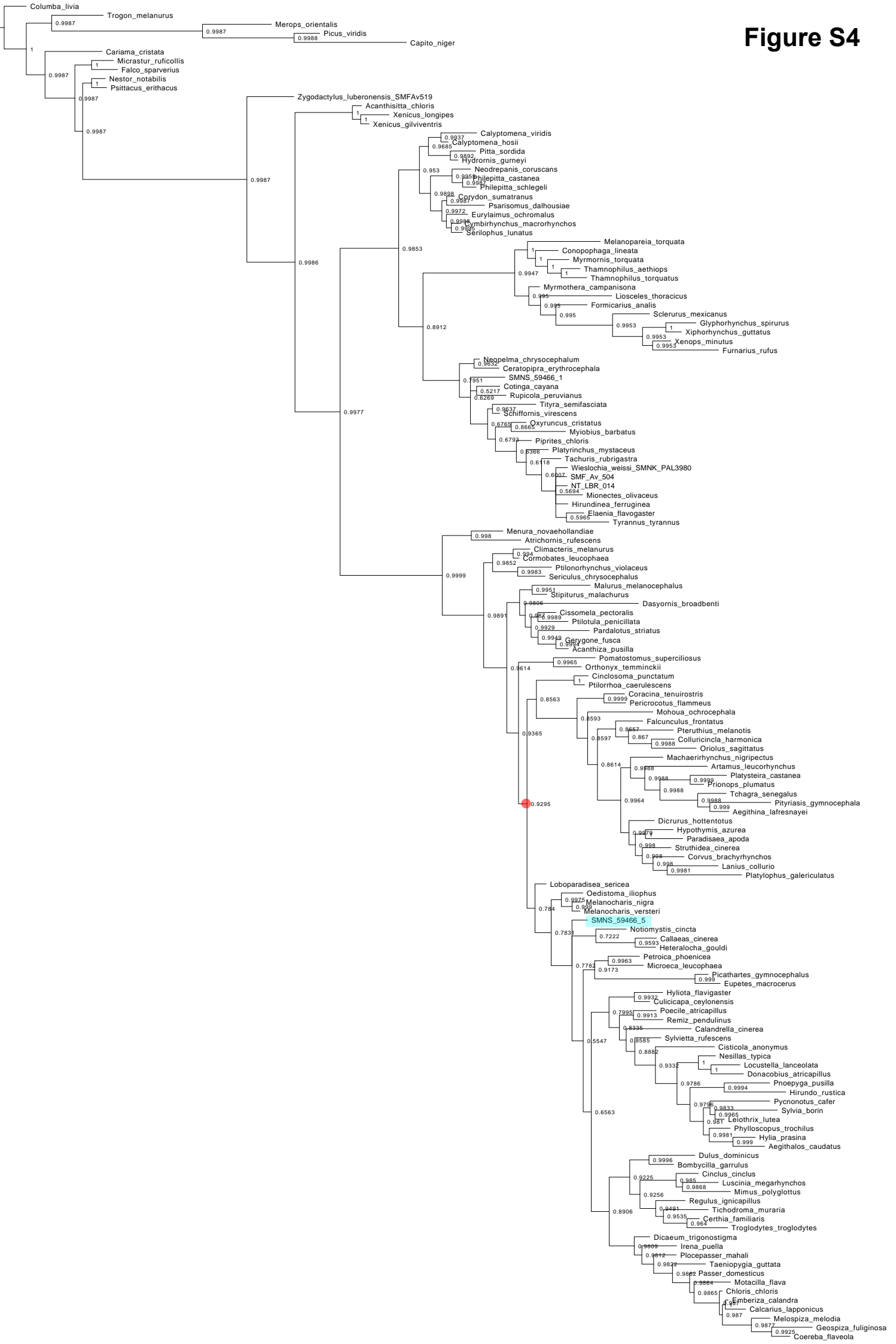

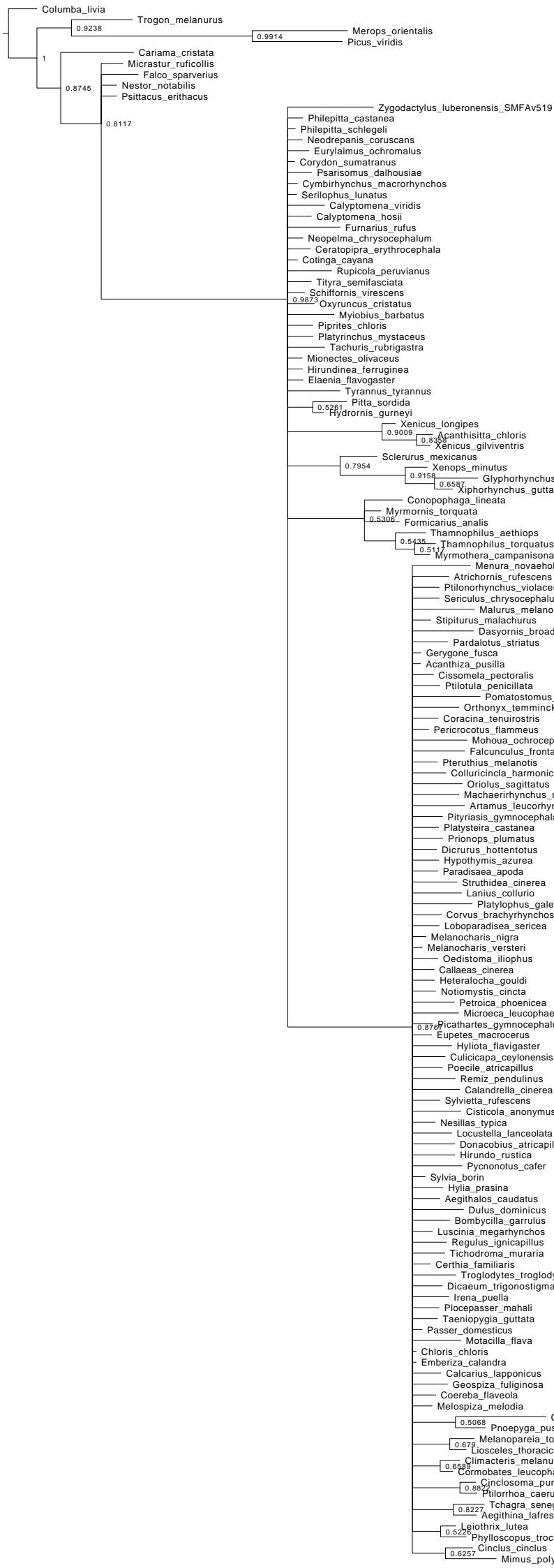

Figure S5

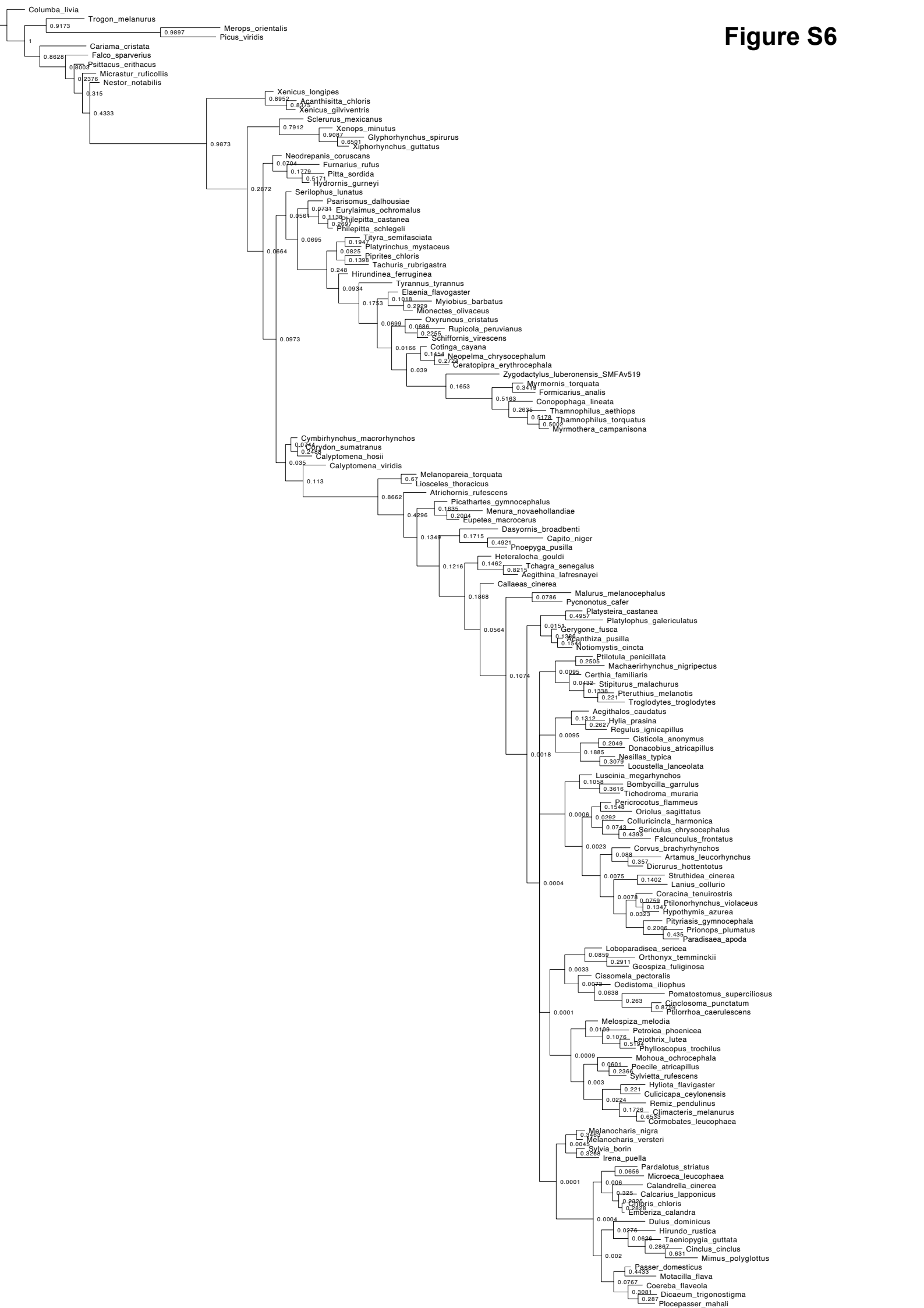

Supplement: Supplementary file 1 — Figures S1‐6 [file JOA-242-495-s005.zip › JOA_13761_SuppInfo_Figures.pdf]
